# Supplementary material for: Genome-Wide Association Study Reveals Novel QTNs and Candidate Genes Implicated in Resistance to Northern Corn Leaf Blight in Maize (Zea mays L.)
Source: Int J Mol Sci. 2025 Nov 2;26(21):10677. doi: 10.3390/ijms262110677 (PMC12608213; doi:10.3390/ijms262110677)
Supplement: Supplementary file 1 [file ijms-26-10677-s001.zip › ijms-3902821-supplementary.pdf]

Supplementary Table S1: Response of inbred lines to Northern corn leaf blight diseases across years

Inbred line	Year	Rep	NCLB score
1	1	1	6
1	1	2	6
1	2	1	5
1	2	2	5
2	1	1	9
2	1	2	9
2	2	1	9
2	2	2	9
3	1	1	8
3	1	2	8
3	2	1	8
3	2	2	8
4	1	1	7
4	1	2	8
4	2	1	7
4	2	2	8
5	1	1	6
5	1	2	5
5	2	1	6
5	2	2	5
6	1	1	5
6	1	2	4
6	2	1	4
6	2	2	4
7	1	1	5
7	1	2	4
7	2	1	4
7	2	2	4
8	1	1	5
8	1	2	5
8	2	1	5
8	2	2	5
9	1	1	4
9	1	2	4
9	2	1	5
9	2	2	4
10	1	1	4
10	1	2	3
10	2	1	4
10	2	2	3
11	1	1	4
11	1	2	3
11	2	1	5

11	2	2	3
12	1	1	4
12	1	2	3
12	2	1	4
12	2	2	5
13	1	1	5
13	1	2	4
13	2	1	4
13	2	2	4
14	1	1	4
14	1	2	4
14	2	1	5
14	2	2	5
15	1	1	5
15	1	2	5
15	2	1	5
15	2	2	4
16	1	1	3
16	1	2	3
16	2	1	2
16	2	2	3
17	1	1	4
17	1	2	4
17	2	1	5
17	2	2	3
18	1	1	5
18	1	2	5
18	2	1	6
18	2	2	5
19	1	1	7
19	1	2	6
19	2	1	7
19	2	2	6
20	1	1	4
20	1	2	4
20	2	1	4
20	2	2	3
21	1	1	5
21	1	2	5
21	2	1	6
21	2	2	5
22	1	1	6
22	1	2	6
22	2	1	5
22	2	2	5
23	1	1	5
23	1	2	5

23	2	1	5
23	2	2	6
24	1	1	4
24	1	2	4
24	2	1	5
24	2	2	5
25	1	1	5
25	1	2	5
25	2	1	4
25	2	2	6
26	1	1	5
26	1	2	5
26	2	1	5
26	2	2	5
27	1	1	7
27	1	2	5
27	2	1	7
27	2	2	6
28	1	1	8
28	1	2	8
28	2	1	8
28	2	2	8
29	1	1	7
29	1	2	6
29	2	1	7
29	2	2	6
30	1	1	7
30	1	2	5
30	2	1	7
30	2	2	6
31	1	1	8
31	1	2	8
31	2	1	8
31	2	2	9
32	1	1	8
32	1	2	8
32	2	1	8
32	2	2	8
33	1	1	7
33	1	2	7
33	2	1	7
33	2	2	7
34	1	1	6
34	1	2	6
34	2	1	6
34	2	2	6
35	1	1	5

35	1	2	5
35	2	1	4
35	2	2	5
36	1	1	5
36	1	2	5
36	2	1	6
36	2	2	5
37	1	1	4
37	1	2	4
37	2	1	4
37	2	2	5
38	1	1	4
38	1	2	5
38	2	1	4
38	2	2	6
39	1	1	4
39	1	2	4
39	2	1	4
39	2	2	6
40	1	1	5
40	1	2	4
40	2	1	6
40	2	2	5
41	1	1	5
41	1	2	5
41	2	1	6
41	2	2	5
42	1	1	6
42	1	2	6
42	2	1	5
42	2	2	6
43	1	1	5
43	1	2	5
43	2	1	5
43	2	2	5
44	1	1	5
44	1	2	5
44	2	1	6
44	2	2	6
45	1	1	4
45	1	2	4
45	2	1	4
45	2	2	4
46	1	1	4
46	1	2	4
46	2	1	5
46	2	2	5

47	1	1	4
47	1	2	4
47	2	1	5
47	2	2	6
48	1	1	4
48	1	2	4
48	2	1	4
48	2	2	4
49	1	1	5
49	1	2	5
49	2	1	6
49	2	2	5
50	1	1	4
50	1	2	4
50	2	1	5
50	2	2	4
51	1	1	5
51	1	2	5
51	2	1	5
51	2	2	6
52	1	1	6
52	1	2	6
52	2	1	7
52	2	2	7
53	1	1	5
53	1	2	5
53	2	1	5
53	2	2	5
54	1	1	6
54	1	2	6
54	2	1	7
54	2	2	6
55	1	1	4
55	1	2	5
55	2	1	4
55	2	2	5
56	1	1	3
56	1	2	3
56	2	1	2
56	2	2	5
57	1	1	4
57	1	2	5
57	2	1	6
57	2	2	5
58	1	1	4
58	1	2	4
58	2	1	5

58	2	2	5
59	1	1	3
59	1	2	3
59	2	1	2
59	2	2	3
60	1	1	3
60	1	2	4
60	2	1	3
60	2	2	4
61	1	1	3
61	1	2	5
61	2	1	3
61	2	2	5
62	1	1	4
62	1	2	4
62	2	1	6
62	2	2	4
63	1	1	5
63	1	2	5
63	2	1	6
63	2	2	5
64	1	1	3
64	1	2	3
64	2	1	2
64	2	2	3
65	1	1	6
65	1	2	6
65	2	1	5
65	2	2	6
66	1	1	4
66	1	2	4
66	2	1	6
66	2	2	5
67	1	1	4
67	1	2	4
67	2	1	5
67	2	2	4
68	1	1	6
68	1	2	5
68	2	1	5
68	2	2	6
69	1	1	5
69	1	2	5
69	2	1	5
69	2	2	6
70	1	1	5
70	1	2	6

70	2	1	6
70	2	2	6
71	1	1	5
71	1	2	5
71	2	1	5
71	2	2	5
72	1	1	3
72	1	2	4
72	2	1	3
72	2	2	6
73	1	1	4
73	1	2	5
73	2	1	4
73	2	2	5
74	1	1	4
74	1	2	4
74	2	1	4
74	2	2	4
75	1	1	3
75	1	2	4
75	2	1	3
75	2	2	4
76	1	1	6
76	1	2	6
76	2	1	7
76	2	2	7
77	1	1	4
77	1	2	4
77	2	1	4
77	2	2	4
78	1	1	5
78	1	2	5
78	2	1	5
78	2	2	5
79	1	1	4
79	1	2	5
79	2	1	4
79	2	2	5
80	1	1	3
80	1	2	4
80	2	1	2
80	2	2	4
81	1	1	5
81	1	2	6
81	2	1	5
81	2	2	6
82	1	1	4

82	1	2	4
82	2	1	4
82	2	2	6
83	1	1	3
83	1	2	3
83	2	1	3
83	2	2	6
84	1	1	4
84	1	2	5
84	2	1	4
84	2	2	6
85	1	1	3
85	1	2	4
85	2	1	2
85	2	2	4
86	1	1	4
86	1	2	5
86	2	1	4
86	2	2	5
87	1	1	3
87	1	2	4
87	2	1	3
87	2	2	4
88	1	1	5
88	1	2	7
88	2	1	5
88	2	2	7
89	1	1	5
89	1	2	5
89	2	1	5
89	2	2	5
90	1	1	3
90	1	2	4
90	2	1	3
90	2	2	4
91	1	1	4
91	1	2	5
91	2	1	4
91	2	2	5
92	1	1	4
92	1	2	5
92	2	1	4
92	2	2	5
93	1	1	5
93	1	2	7
93	2	1	5
93	2	2	9

94	1	1	4
94	1	2	6
94	2	1	4
94	2	2	8
95	1	1	3
95	1	2	4
95	2	1	3
95	2	2	4
96	1	1	4
96	1	2	6
96	2	1	5
96	2	2	6
97	1	1	3
97	1	2	5
97	2	1	3
97	2	2	7
98	1	1	4
98	1	2	6
98	2	1	4
98	2	2	6
99	1	1	3
99	1	2	5
99	2	1	2
99	2	2	5
100	1	1	5
100	1	2	5
100	2	1	5
100	2	2	5
101	1	1	3
101	1	2	7
101	2	1	2
101	2	2	7
102	1	1	4
102	1	2	6
102	2	1	4
102	2	2	6
103	1	1	4
103	1	2	5
103	2	1	5
103	2	2	5
104	1	1	4
104	1	2	6
104	2	1	4
104	2	2	6
105	1	1	3
105	1	2	5
105	2	1	2

105	2	2	5
106	1	1	4
106	1	2	5
106	2	1	4
106	2	2	6
107	1	1	3
107	1	2	4
107	2	1	3
107	2	2	5
108	1	1	2
108	1	2	4
108	2	1	3
108	2	2	5
109	1	1	3
109	1	2	4
109	2	1	4
109	2	2	4
110	1	1	3
110	1	2	3
110	2	1	2
110	2	2	5
111	1	1	4
111	1	2	5
111	2	1	4
111	2	2	5
112	1	1	5
112	1	2	6
112	2	1	5
112	2	2	6
113	1	1	6
113	1	2	7
113	2	1	6
113	2	2	7
114	1	1	6
114	1	2	8
114	2	1	6
114	2	2	8
115	1	1	6
115	1	2	7
115	2	1	6
115	2	2	9
116	1	1	5
116	1	2	6
116	2	1	5
116	2	2	6
117	1	1	6
117	1	2	8

117	2	1	7
117	2	2	8
118	1	1	5
118	1	2	7
118	2	1	7
118	2	2	8
119	1	1	7
119	1	2	8
119	2	1	7
119	2	2	9
120	1	1	6
120	1	2	8
120	2	1	6
120	2	2	8
121	1	1	4
121	1	2	5
121	2	1	4
121	2	2	7
122	1	1	5
122	1	2	6
122	2	1	5
122	2	2	6
123	1	1	6
123	1	2	6
123	2	1	7
123	2	2	6
124	1	1	5
124	1	2	7
124	2	1	5
124	2	2	7
125	1	1	4
125	1	2	6
125	2	1	4
125	2	2	6
126	1	1	4
126	1	2	5
126	2	1	5
126	2	2	5
127	1	1	3
127	1	2	5
127	2	1	3
127	2	2	6
128	1	1	3
128	1	2	5
128	2	1	2
128	2	2	5
129	1	1	4

129	1	2	6
129	2	1	5
129	2	2	6
130	1	1	3
130	1	2	5
130	2	1	2
130	2	2	5
131	1	1	5
131	1	2	6
131	2	1	5
131	2	2	6
132	1	1	3
132	1	2	6
132	2	1	2
132	2	2	6
133	1	1	4
133	1	2	5
133	2	1	4
133	2	2	5
134	1	1	4
134	1	2	6
134	2	1	4
134	2	2	6
135	1	1	4
135	1	2	5
135	2	1	4
135	2	2	5
136	1	1	3
136	1	2	5
136	2	1	3
136	2	2	5
137	1	1	3
137	1	2	4
137	2	1	2
137	2	2	4
138	1	1	3
138	1	2	7
138	2	1	2
138	2	2	7
139	1	1	4
139	1	2	6
139	2	1	4
139	2	2	8
140	1	1	4
140	1	2	5
140	2	1	4
140	2	2	5

141	1	1	4
141	1	2	6
141	2	1	5
141	2	2	6
142	1	1	4
142	1	2	5
142	2	1	4
142	2	2	5
143	1	1	5
143	1	2	7
143	2	1	5
143	2	2	8
144	1	1	4
144	1	2	6
144	2	1	4
144	2	2	6
145	1	1	3
145	1	2	6
145	2	1	4
145	2	2	6
146	1	1	4
146	1	2	7
146	2	1	4
146	2	2	7
147	1	1	3
147	1	2	6
147	2	1	3
147	2	2	6
148	1	1	4
148	1	2	6
148	2	1	4
148	2	2	6
149	1	1	4
149	1	2	6
149	2	1	4
149	2	2	7
150	1	1	3
150	1	2	5
150	2	1	4
150	2	2	5
151	1	1	4
151	1	2	7
151	2	1	5
151	2	2	7
152	1	1	3
152	1	2	6
152	2	1	4

152	2	2	6
153	1	1	4
153	1	2	5
153	2	1	4
153	2	2	7
154	1	1	3
154	1	2	6
154	2	1	2
154	2	2	6
155	1	1	3
155	1	2	6
155	2	1	2
155	2	2	6
156	1	1	4
156	1	2	5
156	2	1	5
156	2	2	5
157	1	1	5
157	1	2	7
157	2	1	5
157	2	2	7
158	1	1	4
158	1	2	6
158	2	1	4
158	2	2	6
159	1	1	4
159	1	2	5
159	2	1	5
159	2	2	5
160	1	1	3
160	1	2	6
160	2	1	4
160	2	2	6
161	1	1	4
161	1	2	5
161	2	1	4
161	2	2	5
162	1	1	4
162	1	2	7
162	2	1	4
162	2	2	7
163	1	1	5
163	1	2	6
163	2	1	5
163	2	2	6
164	1	1	4
164	1	2	5

164	2	1	4
164	2	2	5
165	1	1	5
165	1	2	6
165	2	1	5
165	2	2	8
166	1	1	4
166	1	2	5
166	2	1	4
166	2	2	5
167	1	1	4
167	1	2	6
167	2	1	4
167	2	2	6
168	1	1	3
168	1	2	5
168	2	1	2
168	2	2	5
169	1	1	5
169	1	2	6
169	2	1	5
169	2	2	6
170	1	1	3
170	1	2	5
170	2	1	4
170	2	2	5
171	1	1	3
171	1	2	4
171	2	1	3
171	2	2	5
172	1	1	3
172	1	2	6
172	2	1	2
172	2	2	6
173	1	1	4
173	1	2	5
173	2	1	5
173	2	2	5
174	1	1	3
174	1	2	4
174	2	1	4
174	2	2	5
175	1	1	4
175	1	2	6
175	2	1	5
175	2	2	6
176	1	1	4

176	1	2	5
176	2	1	4
176	2	2	5
177	1	1	3
177	1	2	4
177	2	1	4
177	2	2	4
178	1	1	5
178	1	2	6
178	2	1	5
178	2	2	7
179	1	1	4
179	1	2	6
179	2	1	5
179	2	2	6
180	1	1	6
180	1	2	7
180	2	1	6
180	2	2	7
181	1	1	4
181	1	2	5
181	2	1	4
181	2	2	5
182	1	1	6
182	1	2	8
182	2	1	6
182	2	2	8
183	1	1	5
183	1	2	6
183	2	1	5
183	2	2	6
184	1	1	4
184	1	2	5
184	2	1	4
184	2	2	5
185	1	1	6
185	1	2	7
185	2	1	6
185	2	2	7
186	1	1	4
186	1	2	5
186	2	1	4
186	2	2	5
187	1	1	4
187	1	2	6
187	2	1	5
187	2	2	6

188	1	1	4
188	1	2	8
188	2	1	5
188	2	2	8
189	1	1	3
189	1	2	7
189	2	1	2
189	2	2	7
190	1	1	4
190	1	2	6
190	2	1	4
190	2	2	6
191	1	1	3
191	1	2	5
191	2	1	2
191	2	2	5
192	1	1	3
192	1	2	6
192	2	1	3
192	2	2	6
193	1	1	4
193	1	2	5
193	2	1	4
193	2	2	5
194	1	1	4
194	1	2	5
194	2	1	5
194	2	2	6
195	1	1	5
195	1	2	6
195	2	1	5
195	2	2	6
196	1	1	4
196	1	2	5
196	2	1	4
196	2	2	5
197	1	1	4
197	1	2	5
197	2	1	4
197	2	2	5
198	1	1	4
198	1	2	6
198	2	1	5
198	2	2	6
199	1	1	3
199	1	2	5
199	2	1	2

199	2	2	5
200	1	1	3
200	1	2	4
200	2	1	4
200	2	2	4
201	1	1	4
201	1	2	5
201	2	1	4
201	2	2	5
202	1	1	4
202	1	2	6
202	2	1	4
202	2	2	6
203	1	1	4
203	1	2	5
203	2	1	4
203	2	2	5
204	1	1	4
204	1	2	5
204	2	1	4
204	2	2	5
205	1	1	4
205	1	2	5
205	2	1	4
205	2	2	5
206	1	1	3
206	1	2	6
206	2	1	2
206	2	2	6
207	1	1	3
207	1	2	5
207	2	1	4
207	2	2	5
208	1	1	3
208	1	2	5
208	2	1	5
208	2	2	5
209	1	1	4
209	1	2	6
209	2	1	4
209	2	2	6
210	1	1	4
210	1	2	5
210	2	1	5
210	2	2	5
211	1	1	3
211	1	2	4

211	2	1	4
211	2	2	4
212	1	1	3
212	1	2	6
212	2	1	4
212	2	2	6
213	1	1	4
213	1	2	5
213	2	1	4
213	2	2	5
214	1	1	4
214	1	2	7
214	2	1	4
214	2	2	7
215	1	1	3
215	1	2	5
215	2	1	2
215	2	2	5
216	1	1	4
216	1	2	6
216	2	1	4
216	2	2	6
217	1	1	4
217	1	2	5
217	2	1	4
217	2	2	5
218	1	1	3
218	1	2	4
218	2	1	4
218	2	2	4
219	1	1	6
219	1	2	7
219	2	1	7
219	2	2	7
220	1	1	4
220	1	2	5
220	2	1	4
220	2	2	5
221	1	1	3
221	1	2	5
221	2	1	3
221	2	2	5
222	1	1	3
222	1	2	6
222	2	1	2
222	2	2	6
223	1	1	3

223	1	2	4
223	2	1	3
223	2	2	4
224	1	1	4
224	1	2	5
224	2	1	4
224	2	2	5
225	1	1	4
225	1	2	6
225	2	1	4
225	2	2	6
226	1	1	3
226	1	2	5
226	2	1	4
226	2	2	5
227	1	1	3
227	1	2	4
227	2	1	3
227	2	2	4
228	1	1	5
228	1	2	6
228	2	1	5
228	2	2	6
229	1	1	8
229	1	2	9
229	2	1	8
229	2	2	9
230	1	1	4
230	1	2	6
230	2	1	4
230	2	2	6
231	1	1	4
231	1	2	5
231	2	1	4
231	2	2	5
232	1	1	3
232	1	2	4
232	2	1	4
232	2	2	4
233	1	1	4
233	1	2	6
233	2	1	5
233	2	2	6
234	1	1	5
234	1	2	5
234	2	1	5
234	2	2	5

235	1	1	3
235	1	2	6
235	2	1	2
235	2	2	6
236	1	1	4
236	1	2	5
236	2	1	4
236	2	2	5
237	1	1	4
237	1	2	5
237	2	1	4
237	2	2	5
238	1	1	4
238	1	2	6
238	2	1	4
238	2	2	6
239	1	1	4
239	1	2	5
239	2	1	4
239	2	2	5
240	1	1	4
240	1	2	5
240	2	1	5
240	2	2	5
241	1	1	3
241	1	2	4
241	2	1	3
241	2	2	4
242	1	1	4
242	1	2	5
242	2	1	4
242	2	2	6
243	1	1	5
243	1	2	6
243	2	1	5
243	2	2	6
244	1	1	4
244	1	2	5
244	2	1	4
244	2	2	5
245	1	1	5
245	1	2	6
245	2	1	5
245	2	2	6
246	1	1	4
246	1	2	6
246	2	1	4

246	2	2	6
247	1	1	4
247	1	2	6
247	2	1	4
247	2	2	6
248	1	1	4
248	1	2	5
248	2	1	5
248	2	2	5
249	1	1	3
249	1	2	4
249	2	1	3
249	2	2	4
250	1	1	5
250	1	2	6
250	2	1	5
250	2	2	6
251	1	1	6
251	1	2	7
251	2	1	6
251	2	2	7
252	1	1	4
252	1	2	5
252	2	1	4
252	2	2	5
253	1	1	3
253	1	2	5
253	2	1	2
253	2	2	5
254	1	1	3
254	1	2	4
254	2	1	3
254	2	2	4
255	1	1	4
255	1	2	5
255	2	1	4
255	2	2	5
256	1	1	5
256	1	2	6
256	2	1	5
256	2	2	6
257	1	1	4
257	1	2	5
257	2	1	4
257	2	2	5
258	1	1	3
258	1	2	4

258	2	1	2
258	2	2	4
259	1	1	4
259	1	2	5
259	2	1	4
259	2	2	5
260	1	1	3
260	1	2	4
260	2	1	4
260	2	2	4
261	1	1	4
261	1	2	5
261	2	1	4
261	2	2	5
262	1	1	3
262	1	2	4
262	2	1	3
262	2	2	4
263	1	1	5
263	1	2	6
263	2	1	5
263	2	2	6
264	1	1	4
264	1	2	6
264	2	1	4
264	2	2	6
265	1	1	4
265	1	2	5
265	2	1	4
265	2	2	5
266	1	1	4
266	1	2	5
266	2	1	4
266	2	2	5
267	1	1	4
267	1	2	5
267	2	1	4
267	2	2	5
268	1	1	3
268	1	2	4
268	2	1	3
268	2	2	4
269	1	1	4
269	1	2	5
269	2	1	4
269	2	2	5
270	1	1	5

270	1	2	6
270	2	1	5
270	2	2	6
271	1	1	4
271	1	2	5
271	2	1	4
271	2	2	5
272	1	1	3
272	1	2	4
272	2	1	4
272	2	2	4
273	1	1	4
273	1	2	4
273	2	1	4
273	2	2	4
274	1	1	4
274	1	2	5
274	2	1	6
274	2	2	5
275	1	1	3
275	1	2	4
275	2	1	3
275	2	2	4
276	1	1	4
276	1	2	5
276	2	1	4
276	2	2	5
277	1	1	3
277	1	2	5
277	2	1	2
277	2	2	5
278	1	1	3
278	1	2	5
278	2	1	2
278	2	2	5
279	1	1	4
279	1	2	4
279	2	1	4
279	2	2	3
280	1	1	3
280	1	2	5
280	2	1	2
280	2	2	5
281	1	1	3
281	1	2	4
281	2	1	2
281	2	2	4

282	1	1	3
282	1	2	5
282	2	1	3
282	2	2	5
283	1	1	3
283	1	2	4
283	2	1	4
283	2	2	4
284	1	1	4
284	1	2	5
284	2	1	4
284	2	2	5
285	1	1	3
285	1	2	4
285	2	1	3
285	2	2	4
286	1	1	6
286	1	2	7
286	2	1	7
286	2	2	7
287	1	1	3
287	1	2	4
287	2	1	2
287	2	2	4
288	1	1	4
288	1	2	5
288	2	1	4
288	2	2	6
289	1	1	4
289	1	2	5
289	2	1	5
289	2	2	5
290	1	1	3
290	1	2	5
290	2	1	2
290	2	2	6
291	1	1	3
291	1	2	4
291	2	1	4
291	2	2	5
292	1	1	3
292	1	2	5
292	2	1	4
292	2	2	4
293	1	1	4
293	1	2	5
293	2	1	4

293	2	2	5
294	1	1	3
294	1	2	4
294	2	1	3
294	2	2	4
295	1	1	3
295	1	2	4
295	2	1	4
295	2	2	3
296	1	1	5
296	1	2	5
296	2	1	5
296	2	2	5
297	1	1	3
297	1	2	4
297	2	1	3
297	2	2	4
298	1	1	3
298	1	2	4
298	2	1	5
298	2	2	3
299	1	1	5
299	1	2	6
299	2	1	5
299	2	2	5
300	1	1	4
300	1	2	5
300	2	1	5
300	2	2	5
301	1	1	4
301	1	2	5
301	2	1	5
301	2	2	5
302	1	1	4
302	1	2	5
302	2	1	4
302	2	2	5
303	1	1	4
303	1	2	5
303	2	1	5
303	2	2	5
304	1	1	3
304	1	2	4
304	2	1	3
304	2	2	4
305	1	1	4
305	1	2	5

305	2	1	4
305	2	2	5
306	1	1	4
306	1	2	5
306	2	1	4
306	2	2	5
307	1	1	3
307	1	2	4
307	2	1	5
307	2	2	4
308	1	1	4
308	1	2	5
308	2	1	5
308	2	2	5
309	1	1	3
309	1	2	4
309	2	1	3
309	2	2	4
310	1	1	4
310	1	2	4
310	2	1	4
310	2	2	4
311	1	1	4
311	1	2	4
311	2	1	4
311	2	2	4
312	1	1	3
312	1	2	4
312	2	1	2
312	2	2	4
313	1	1	3
313	1	2	4
313	2	1	3
313	2	2	4
314	1	1	4
314	1	2	5
314	2	1	4
314	2	2	5
315	1	1	3
315	1	2	4
315	2	1	4
315	2	2	3
316	1	1	4
316	1	2	5
316	2	1	4
316	2	2	5
317	1	1	3

317	1	2	4
317	2	1	3
317	2	2	4
318	1	1	4
318	1	2	4
318	2	1	4
318	2	2	3
319	1	1	3
319	1	2	4
319	2	1	2
319	2	2	4
320	1	1	5
320	1	2	5
320	2	1	5
320	2	2	5
321	1	1	4
321	1	2	4
321	2	1	4
321	2	2	4
322	1	1	3
322	1	2	3
322	2	1	2
322	2	2	4
323	1	1	3
323	1	2	4
323	2	1	3
323	2	2	5
324	1	1	3
324	1	2	4
324	2	1	3
324	2	2	3
325	1	1	4
325	1	2	5
325	2	1	4
325	2	2	5
326	1	1	5
326	1	2	4
326	2	1	5
326	2	2	4
327	1	1	5
327	1	2	5
327	2	1	5
327	2	2	5
328	1	1	4
328	1	2	5
328	2	1	4
328	2	2	4

329	1	1	3
329	1	2	4
329	2	1	4
329	2	2	5
330	1	1	3
330	1	2	4
330	2	1	3
330	2	2	4
331	1	1	4
331	1	2	3
331	2	1	4
331	2	2	4
332	1	1	6
332	1	2	5
332	2	1	6
332	2	2	5
333	1	1	3
333	1	2	4
333	2	1	4
333	2	2	4
334	1	1	8
334	1	2	9
334	2	1	8
334	2	2	9
335	1	1	4
335	1	2	5
335	2	1	4
335	2	2	4
336	1	1	3
336	1	2	4
336	2	1	2
336	2	2	5

Supplementary Table S2: Identified QTNs, their positions, effects and PVE explained

SNP	Chromosome	Position	P.value	maf	nobs	effect	PVE (%)	GWAS model
S1_5517184	1	5517184	8.62E-09	0.072413793	290	-0.636974866	6.369138694	GLM
S1_7162498	1	7162498	5.22E-09	0.177586207	290	-0.585920933	0.445311483	GLM
S1_7356398	1	7356398	4.35E-11	0.062068966	290	-0.785397659	12.12356702	GLM
S1_82025948	1	82025948	4.98E-08	0.070689655	290	-0.632061048	1.080370692	GLM
S2_13448516	2	13448516	4.93E-08	0.156896552	290	-0.488599597	1.964524605	GLM
S2_22706963	2	22706963	5.66E-08	0.139655172	290	-0.505750823	0.525872767	GLM
S2_41007941	2	41007941	6.32E-08	0.139655172	290	-0.466735808	0.169192094	GLM
S2_54296437	2	54296437	1.73E-07	0.163793103	290	-0.405714322	0.741990883	GLM
S2_68998339	2	68998339	2.93E-09	0.25862069	290	-0.438523232	2.603793333	GLM
S3_148220101	3	148220101	4.42E-08	0.05862069	290	-0.764715082	1.56E-07	GLM
S3_159685959	3	159685959	1.34E-07	0.118965517	290	-0.441631491	6.94E-05	GLM
S3_164976764	3	164976764	5.71E-08	0.072413793	290	-0.64248947	0.079442977	GLM
S3_218006371	3	218006371	3.27E-09	0.1	290	-0.666084603	5.977854619	GLM
S3_219836030	3	219836030	4.48E-09	0.056896552	290	-0.724252617	9.54E-09	GLM
S3_219855710	3	219855710	5.17E-08	0.1	290	-0.541255084	3.437988037	GLM
S3_219868986	3	219868986	3.40E-08	0.068965517	290	-0.672997018	0.729734458	GLM
S4_2653687	4	2653687	1.62E-07	0.105172414	290	-0.491184867	0.6	GLM
S5_952051	5	952051	5.40E-10	0.089655172	290	-0.61565299	0.35008	GLM
S5_1185116	5	1185116	1.48E-09	0.084482759	290	-0.635758362	7.92E-09	GLM
S5_3396708	5	3396708	9.16E-09	0.074137931	290	-0.604244045	0.06688849	GLM
S5_3412198	5	3412198	1.08E-07	0.094827586	290	-0.563671861	3.58E-07	GLM
S5_3428332	5	3428332	3.02E-09	0.05	290	-0.697208658	3.65E-08	GLM
S5_3428378	5	3428378	4.02E-09	0.051724138	290	-0.684497586	1.43E-07	GLM
S5_3544603	5	3544603	1.42E-08	0.086206897	290	-0.548045566	6.499801092	GLM
S5_4321966	5	4321966	1.26E-08	0.056896552	290	-0.661137104	1.43E-07	GLM
S5_4469882	5	4469882	1.45E-07	0.196551724	290	-0.379937692	0.603794823	GLM
S5_4469928	5	4469928	1.45E-07	0.196551724	290	-0.379937692	0.002047247	GLM

S5_4780212	5	4780212	2.24E-08	0.086206897	290	-0.597331505	1.57E-08	GLM
S5_5749988	5	5749988	2.40E-08	0.070689655	290	-0.567654367	5.50E-08	GLM
S5_5781412	5	5781412	5.20E-08	0.075862069	290	-0.546911921	0.002589	GLM
S5_7573553	5	7573553	1.11E-07	0.055172414	290	-0.657664382	6.76E-09	GLM
S5_21933117	5	21933117	1.30E-08	0.131034483	290	-0.478881969	1.5490058	GLM
S5_205748142	5	205748142	3.72E-09	0.084482759	290	-0.64969777	2.638784232	GLM
S5_209255165	5	209255165	1.26E-07	0.060344828	290	-0.626578256	1.57E-08	GLM
S5_209467941	5	209467941	7.88E-08	0.055172414	290	-0.639196869	1.38E-07	GLM
S5_212349596	5	212349596	5.27E-08	0.074137931	290	-0.611010503	1.14E-08	GLM
S5_212349600	5	212349600	6.90E-08	0.079310345	290	-0.579049112	0.001485	GLM
S5_212874755	5	212874755	1.38E-08	0.105172414	290	-0.566607618	0.1285	GLM
S5_212874756	5	212874756	1.38E-08	0.105172414	290	-0.566607618	5.50E-08	GLM
S5_212874757	5	212874757	1.38E-08	0.105172414	290	-0.566607618	0.5286	GLM
S5_213038514	5	213038514	2.80E-08	0.112068966	290	-0.492624794	0.33688	GLM
S5_213038575	5	213038575	5.75E-10	0.075862069	290	-0.645846786	0.44789	GLM
S5_213038576	5	213038576	5.75E-10	0.075862069	290	-0.645846786	0.014478	GLM
S5_216545141	5	216545141	4.15E-08	0.079310345	290	-0.560217932	0.22535	GLM
S6_36624158	6	36624158	4.45E-09	0.063793103	290	-0.657149809	1.26E-07	GLM
S6_47308425	6	47308425	1.45E-08	0.05862069	290	-0.620429919	8.57E-09	GLM
S6_147920879	6	147920879	6.58E-08	0.065517241	290	-0.612571471	0.840552779	GLM
S7_137512822	7	137512822	7.49E-08	0.136206897	290	-0.522786205	0.2358	GLM
S7_142113656	7	142113656	6.08E-08	0.263793103	290	-0.326576519	0.66292963	GLM
S7_142113657	7	142113657	9.68E-08	0.267241379	290	-0.319971925	1.09E-07	GLM
S7_142113659	7	142113659	6.08E-08	0.263793103	290	-0.326576519	0.36090932	GLM
S7_145081423	7	145081423	5.05E-09	0.379310345	290	-0.338098923	2.711530802	GLM
S7_161349276	7	161349276	1.26E-09	0.081034483	290	-0.721835489	9.06E-07	GLM
S7_172659333	7	172659333	4.90E-08	0.065517241	290	-0.590525411	3.430161286	GLM
S7_174501701	7	174501701	4.06E-09	0.10862069	290	-0.591584446	1.844506752	GLM
S7_174717937	7	174717937	1.04E-07	0.086206897	290	-0.633686763	1.2258	GLM
S9_57187283	9	57187283	1.23E-07	0.143103448	290	-0.450597672	2.657505511	GLM

S9_57187484	9	57187484	1.30E-07	0.137931034	290	-0.441122283	4.06E-07	GLM
S9_69968922	9	69968922	5.23E-09	0.137931034	290	-0.519025096	4.287647461	GLM
S9_71187867	9	71187867	3.18E-09	0.132758621	290	-0.520152616	0.14889	GLM
S9_99293080	9	99293080	3.14E-08	0.396551724	290	-0.374035724	5.213676853	GLM
S9_118388897	9	118388897	1.00E-07	0.05862069	290	-0.612968402	4.29E-08	GLM
S9_118923655	9	118923655	9.87E-09	0.062068966	290	-0.693904032	0.12589	GLM
S9_119286841	9	119286841	2.54E-09	0.053448276	290	-0.741942798	4.83E-08	GLM
S9_120645995	9	120645995	1.13E-07	0.087931034	290	-0.581683181	7.29E-08	GLM
S9_128539160	9	128539160	5.04E-09	0.139655172	290	-0.630138015	0.14789	GLM
S9_128539163	9	128539163	3.52E-09	0.136206897	290	-0.643214558	6.87E-07	GLM
S9_143203280	9	143203280	4.71E-08	0.103448276	290	-0.518391735	3.071358587	GLM
S9_151993415	9	151993415	1.62E-07	0.232758621	290	-0.363738099	1.152907967	GLM
S9_153511132	9	153511132	5.71E-09	0.055172414	290	-0.712184168	8.22E-09	GLM
S1_290805849	1	290805849	9.90E-10	0.134482759	290	-0.300988317	4.616173563	FarmCPU
S2_51098833	2	51098833	1.17E-07	0.320689655	290	-0.166505844	6.105855561	FarmCPU
S3_57083345	3	57083345	1.05E-08	0.213793103	290	0.240478555	0.342999658	FarmCPU
S8_65046664	8	65046664	8.79E-09	0.168965517	290	-0.221801247	0.223	FarmCPU
